# Coronavirus disease 2019 (COVID-19) Brazil Task Force: How to navigate troubled waters

**DOI:** 10.1017/ice.2021.41

**Published:** 2021-02-02

**Authors:** Marcelo Carneiro, Viviane Maria de Carvalho Hessel Dias, Magda Machado de Miranda Costa, Débora Otero Britto Passos Pinheiro, Cláudia Fernanda de Lacerda Vidal, Olívia Cristina Palmeira da Silva Rodrigues, Mirian de Freitas Dal Ben Corradi, Heiko Thereza Santana, Maria Dolores Santos da Purificação Nogueira, Mara Rúbia Santos Gonçalves, Maria Clara Padoveze

**Affiliations:** 1 Brazilian Association of Hospital Infection Control and Epidemiology (ABIH), Curitiba, Brazil; 2 General Management of Technology in Health Services (GGTES), Brazilian Health Regulatory Agency (ANVISA), Brasilia, Brazil

*To the Editor—*The coronavirus disease 2019 (COVID-19) pandemic has required responses from national and international authorities, scientific societies, workers’ unions, healthcare professional councils, organizations at multisector areas, and the entire population to apply strategies aimed at disease control and prevention. Meanwhile, these organizations face many associated political and social challenges.^1^

Brazil is a country with marked social inequality and sociocultural heterogeneity. To face the COVID-19 crisis, this context demands regionalized actions, as well as availability and effective management of financial resources. The country has one of the largest universal public health systems in the world, the Brazilian Unified Health System, which is fully funded and articulated at the 3 levels of government levels (federal, state, and municipal). However, strong restriction of funding has occurred since 2016,^2^ and under the current emergency situation, the public health system could collapse, leading to increased mortality due to lack of hospital beds and intensive care.^3^ Thus, several vulnerabilities of the system have been exposed, similar to what has occurred in other countries.^4^

The first documented case of COVID-19 in Brazil was detected on February 26, 2020, in the city of São Paulo. By March 20, community transmission status was declared in the country.^5^ Since then, concerns related to the occupational risk of healthcare workers has propelled a movement of workers’ unions and professional societies and councils, leading to a myriad of published documents with diverse and often conflicting recommendations, especially regarding the use of personal (PPE) and collective protective equipment. These contradicting directions have fostered a sense of insecurity among healthcare professionals and have increased the challenges for infection prevention and control personnel already overwhelmed by the excessive workload.

The Brazilian Association of Hospital Infection Control and Epidemiology (ABIH) has aimed to achieve a technical consensus among stakeholders. The ABIH is a scientific, nonprofit society that connects professionals who work in infection control in the country by coordinating publications in the area and collaborating with academic organizations to disseminate evidence-based information.

In line with its essential purpose, the ABIH has led an initiative to assemble specialists from different areas of medicine and nursing and representatives from the Brazilian Health Regulatory Agency (ANVISA), to promote an interdisciplinary debate that will build a technical-scientific consensus to guide and assist health workers in understanding, managing, and coping with the COVID-19 pandemic. This group is called the “COVID-19 Brazil Task Force,” and meetings have been held weekly online.

To date, 4 documents have been developed by this task force and have been published by ANVISA: (1) Technical note on the guidelines for health services: prevention and control measures that must be adopted when assisting suspected or confirmed cases of infection by the new coronavirus; (2) technical note on the guidelines for the prevention and control of infections by the new coronavirus in surgical procedures; (3) a review of all the scientific evidence accumulated to date on COVID-19; and (4) guidelines for preventing the transmission of COVID-19 in the health services.^6–9^

However, we are aware that the problems related to the structure, training, and monitoring in health institutions have been a constant challenge in most hospitals, mainly those with already insufficient structure.^10^ These challenges include the shortage of PPE in some regions of the country, which has led to a market of low-quality products and increased hospital costs. To minimize the problem, community initiatives have been undertaken to build networks to assist health professionals and reduce the financial impact of the pandemic by offering low-cost technologies and donations. These resources are channeled by the ABIH to the healthcare settings that need them the most.

The COVID-19 Brazil Task Force has yielded 3 main benefits. First, it unifies several societies toward a common objective, leaving aside differences and contributing to the construction of multidisciplinary guidance to support the frontline health workers. Second, this initiative has enlarged the channels of dissemination of guidance related to COVID-19 prevention and control, which will contribute to greater adherence. Third, this low-cost initiative may become an example for similar future cooperation related to other challenges of common interest, such as antimicrobial resistance.

As an example of future cooperation, we appreciate that the task force has valued teamwork among the diverse specialties involved. Despite the current challenges in Brazilian health care, this group of devoted professionals have focused on finding ways to meet them.

## References

[1] Darsie C , Weber DL. Disease and space control: issues about dispersion and isolation in pandemic times? J Infect Control 2020;9:47–48.

[2] GBD 2016 Brazil Collaborators. Burden of disease in Brazil, 1990–2016: a systematic subnational analysis for the Global Burden of Disease Study 2016. Lancet 2018;392:75–76. 30037735 10.1016/S0140-6736(18)31221-2 PMC6123514

[3] Oliveira WK , Duarte E , França GVA , Garcia LP. How Brazil can hold back COVID-19. Epidemiol Serv Saude 2020;29:e2020044. 32348405 10.5123/s1679-49742020000200023

[4] India under COVID-19 lockdown. *Lancet* 2020;395:1315. 10.1016/S0140-6736(20)30938-7 PMC7180023 32334687

[5] Brasil Ministerio de Saude website. https://www.saude.gov.br/noticias/agencia-saude/46568-ministerio-da-saude-declara-transmissao-comunitaria-nacional. Updated 2020. Accessed February 1, 2021.

[6] Nota técnica Covid-19 (No. 4). ANVISA website. https://www20.anvisa.gov.br/segurancadopaciente/index.php/alertas/item/nota-tecnica?category_id=244. Updated 2020. Accessed February 1, 2021.

[7] Nota técnica Covid-19 (No. 5). ANVISA website. https://www20.anvisa.gov.br/segurancadopaciente/index.php/alertas/item/nota-tecnica-gvims-ggtes-anvisa-n-07-2021?category_id=244. Updated 2020. Accessed February 1, 2021.

[8] Nota técnica Covid-19 (No. 7). ANVISA website. https://www20.anvisa.gov.br/segurancadopaciente/index.php/alertas/item/nota-tecnica-gvims-ggtes-anvisa-n-07-2020?category_id=244. Updated 2020. Accessed February 1, 2021.

[9] de Carvalho Hessel Dias VM , Carneiro M , de Lacerda Vidal CF , et al. Guidelines on diagnosis, treatment and isolation of patients with COVID-19. J Infect Control 2020;9:56–75.

[10] Padoveze MC , Fortaleza CMCB , Kiffer C , Carneiro ICRS , Barth AL , Giamberardino HIG , et al. Structure for prevention of health care-associated infections in Brazilian hospitals: a countrywide study. Am J Infect Control 2016;44:74–79. 26412480 10.1016/j.ajic.2015.08.004

